# Residential Exposure to Natural Background Radiation and Risk of Childhood Acute Leukemia in France, 1990–2009

**DOI:** 10.1289/EHP296

**Published:** 2016-08-02

**Authors:** Claire Demoury, Fabienne Marquant, Géraldine Ielsch, Stéphanie Goujon, Christophe Debayle, Laure Faure, Astrid Coste, Olivier Laurent, Jérôme Guillevic, Dominique Laurier, Denis Hémon, Jacqueline Clavel

**Affiliations:** 1 INSERM, Université Paris-Descartes, Université Sorbonne-Paris-Cité, CRESS-EPICEA Epidémiologie des cancers de l’enfant et de l’adolescent, Paris, France; 2 Institut de Radioprotection et de Sûreté Nucléaire (IRSN), Bureau d’étude et d’expertise du radon et de la modélisation (PRP-DGE/SEDRAN/BERAM), Fontenay aux Roses, France; 3 French National Registry of Childhood Hematological Malignancies, Villejuif, France; 4 IRSN, Laboratoire de surveillance atmosphérique et d’alerte (PRP-ENV/SESURE/LS2A), Le Vésinet, France; 5 IRSN, Laboratoire d’épidémiologie des rayonnements ionisants (PRP-HOM/SRBE/LEPID), Fontenay aux Roses, France

## Abstract

**Background:**

Exposures to high-dose ionizing radiation and high-dose rate ionizing radiation are established risk factors for childhood acute leukemia (AL). The risk of AL following exposure to lower doses due to natural background radiation (NBR) has yet to be conclusively determined.

**Methods:**

AL cases diagnosed over 1990–2009 (9,056 cases) were identified and their municipality of residence at diagnosis collected by the National Registry of Childhood Cancers. The Geocap study, which included the 2,763 cases in 2002–2007 and 30,000 population controls, was used for complementary analyses. NBR exposures were modeled on a fine scale (36,326 municipalities) based on measurement campaigns and geological data. The power to detect an association between AL and dose to the red bone marrow (RBM) fitting UNSCEAR (United Nations Scientific Committee on the Effects of Atomic Radiation) predictions was 92%, 45% and 99% for exposure to natural gamma radiation, radon and total radiation, respectively.

**Results:**

AL risk, irrespective of subtype and age group, was not associated with the exposure of municipalities to radon or gamma radiation in terms of yearly exposure at age reached, cumulative exposure or RBM dose. There was no confounding effect of census-based socio-demographic indicators, or environmental factors (road traffic, high voltage power lines, vicinity of nuclear plants) related to AL in the Geocap study.

**Conclusions:**

Our findings do not support the hypothesis that residential exposure to NBR increases the risk of AL, despite the large size of the study, fine scale exposure estimates and wide range of exposures over France. However, our results at the time of diagnosis do not rule out a slight association with gamma radiation at the time of birth, which would be more in line with the recent findings in the UK and Switzerland.

**Citation:**

Demoury C, Marquant F, Ielsch G, Goujon S, Debayle C, Faure L, Coste A, Laurent O, Guillevic J, Laurier D, Hémon D, Clavel J. 2017. Residential exposure to natural background radiation and risk of childhood acute leukemia in France, 1990–2009. Environ Health Perspect 125:714–720; http://dx.doi.org/10.1289/EHP296

## Introduction

Ionizing radiation is an established risk factor for childhood acute leukemia (AL). This has been demonstrated by several studies, including the follow-up of survivors of the Hiroshima and Nagasaki atomic bombs (Hsu et al. 2013; Preston et al. 1994), studies of populations exposed therapeutically to radiation (Pearce et al. 2012; UNSCEAR 2000), and studies of populations exposed *in utero* (Doll and Wakeford 1997; Wakeford and Little 2003). The literature was recently extensively reviewed (Wakeford 2013). The United Nations Scientific Committee on the Effects of Atomic Radiation (UNSCEAR 2006) and the Committee of the National Research Council of the United States on the Biological Effects of Ionizing Radiation (BEIR) (NRC 2006) have developed risk models that can be used to estimate leukemia risk associated with different patterns of radiation exposure (chronic or acute, external or internal).

Natural background radiation (NBR) leads to low-dose rates of exposure in the population. NBR comprises external exposure to cosmic and terrestrial gamma radiation and internal exposure by inhalation of radon gas and ingestion of radionuclides (UNSCEAR 2000). While dose from radon and its decay products is primarily delivered to the respiratory system, doses from terrestrial gamma and cosmic radiation are more homogeneously delivered throughout the body, including to the red bone marrow (RBM) (Kendall et al. 2009), which is thought to be the primary site of leukemia initiation.

Since the late 1980s, ecological studies in many countries have linked large-scale geographic variations in the incidence of AL with variations in average radon and gamma radiation exposure on that scale. As stated in several reviews (Laurier et al. 2001; Raaschou-Nielsen 2008; Tong et al. 2012), most of the studies found positive correlations with radon, whereas no evidence was found for gamma radiation exposure. Population-based case–control studies that included interviews and in-home measurements have reported mixed results, mostly nonsignificant (Raaschou-Nielsen 2008), but their interpretation is hindered by the generally limited participation rates and the subsequent potential for selection bias. More recently, record-based studies were developed on a nationwide scale in the general population, using modeled estimates of exposure to ionizing radiation and the geolocation of homes. A Danish record-based case–control study (Raaschou-Nielsen et al. 2008), which included 860 cases of AL, found an association between lifelong domestic radon exposure and AL incidence rates. Two population-based record-based cohort studies, a Swiss cohort that included 149 cases (Hauri et al. 2013) and a Norwegian cohort that included 437 cases (Del Risco Kollerud et al. 2014), did not find any association with radon exposure. In the United Kingdom, a population-based record-based case–control study that compared 9,058 AL cases and 11,912 controls showed an association with gamma radiation but not with radon at the residence at birth (Kendall et al. 2013). The expanded Swiss cohort (530 AL cases) study found a statistically significant association between estimated cumulative exposure to gamma radiation and AL (Spycher et al. 2015).

Using the UNSCEAR 2006 radiation risk models (UNSCEAR 2006), the attributable fraction of AL risk related to NBR was estimated to be 15–20% in the United Kingdom (Little et al. 2009). In France, the estimated attributable fractions of cases associated with radon, terrestrial gamma radiation, and cosmic rays were 20% (95% credible interval: 0–68%) under an excess relative risk model (ERR) and 4% (95% credible interval: 0–11%) under an excess absolute risk model, considering uncertainties in radiation-related leukemia risk model parameters within a Bayesian framework (Laurent et al. 2013).

The direct observation of the association between AL incidence rates and home location–based estimated exposure to NBR has not provided fully coherent qualitative and quantitative results, even in the recent nationwide record-based studies.

The present study investigated the association in France over the period 1990–2009 (9,056 AL cases) using the data from the French National Registry of Childhood Cancer (RNCE) and the precise local information on exposure to NBR modeled on the scale of the 36,326 French municipalities. In addition, we also analyzed the exposure to NBR in the Geocap population-based record-based study (2,763 AL cases and 30,000 controls with geocoded addresses) over 2002–2007.

## Methods

### Population

***Incidence study 1990–2009.*** The study included all the cases of AL diagnosed between 1 January 1990, and 31 December 2009, in children < 15 years old and living in mainland France at the time of diagnosis. The cases were identified by the RNCE (Clavel et al. 2004; Lacour et al. 2010).

The population data from 1990 to 2009 were estimated from census data provided by the French National Institute for Statistics and Economic Studies (INSEE) and the yearly nationwide age- and gender-specific incidence rates were provided by the RNCE. They were used to derive annual expected numbers of cases by 1-year age group and gender for all the municipalities (i.e., Communes), which are the smallest French administrative units. A few municipalities were grouped together to account for changes in their perimeters over the study period.

***Geocap case–control study 2002–2007.*** In addition to the incidence study, we used the Geocap study, a population-based record-based case–control study conducted over the period 2002–2007. The study included all the cases diagnosed during that period and 30,000 contemporaneous control addresses (5,000 per year) randomly sampled by the INSEE using the income and council tax databases of the households. The controls were closely representative of the French residents < 15 years old in terms of age and number of children in the household, and in terms of the socioeconomic and demographic characteristics of the municipality of residence [size of the urban unit, median income, proportion of blue-collar workers, proportion of subjects who successfully completed high school (baccalaureate holders), and proportion of homeowners] (Houot et al. 2015; Sermage-Faure et al. 2012).

### Exposure Assessment

The municipalities and geocoded addresses of the residences were available at the time of diagnosis (cases, 1990–2009) or inclusion (controls, 2002–2007). They were geocoded using the MapInfo GIS (version 8.5; Pitney Bowes Software Inc., Troy, New York, USA), 2010 street databases (NAVTEQ, Paris, France), and detailed vectorized maps from the Institut national de l’information géographique et forestière. NBR exposures were determined at the town center for radon, as the mean exposure over the municipality territory for gamma, in the incidence study and at the residence address and the town center in the case–control study.

***Exposure to radon.*** The radon domestic exposure was estimated from two data sets: 10,843 measurement results of indoor radon concentration performed by the Institute for Radiological Protection and Nuclear Safety (IRSN) and the Health Ministry (DGS, Direction Générale de la Santé) during a national campaign (1982–2003) (see Supplemental Material, “Part 1”), and the French map of the geogenic radon potential (Ielsch et al. 2010). A cokriging model (Wackernagel 2013) using both data sets was developed and provided estimates of the indoor radon concentrations resulting in a 1 × 1 km^2^ grid (see Supplemental Material, “Part 1”) (IRSN Report 2012). Based on this modeling approach, 32% of the variance of individual radon exposure measurements was explained by the spatial coordinates of the home location (IRSN Report 2012).

***Exposure to gamma radiation.*** Exposure to natural gamma radiation was calculated as the sum of exposure to cosmic gamma radiation and terrestrial gamma radiation. The latter was determined using a method recently proposed by Warnery et al. (2015). The determination was based on 97,595 measurement results of indoor gamma dose rate conducted by the IRSN in 17,404 dental surgeries and veterinary clinics throughout France. The IRSN used radio photoluminescent (RPL) dosimeters, exposed for several months in 2011–2012 (see Supplemental Material, “Part 1”), from which the cosmic component, which was estimated using the UNSCEAR formula (UNSCEAR 2000), was first subtracted. To estimate the indoor telluric gamma dose rate, multi-collocated cokriging was conducted on a 1 × 1 km^2^ grid in a geostatistical model that used two data sets: the indoor terrestrial gamma radiation dose rate measurement results and the French map of geogenic uranium potential (Ielsch et al. 2016) (see Supplemental Material, “Part 1”). Based on this modeling approach, 65% of the variance of the indoor terrestrial gamma radiation measurements was explained by the spatial coordinates of the home location (Warnery et al. 2015). The cosmic component of gamma radiation exposure was calculated for each location based on the UNSCEAR formula (UNSCEAR 2000) and then added to the terrestrial gamma radiation exposure to obtain total gamma radiation exposure.

***Cumulative exposure and cumulative RBM dose.*** Since residential histories were not available in the study, cumulative external exposures to radon (Bq/m^3^ × year) or gamma radiation (mSv × year) were extrapolated from local estimates of exposure at diagnosis or inclusion, assuming that the same exposure had prevailed since birth. In the ESCALE (Etude Sur les Cancers et les Leucémies de l’Enfant, Study on Environmental and Genetic Risk Factors of Childhood Cancers and Leukemia) interview-based study (Rudant et al. 2007), 66% of the children had been living in the same municipality since birth, and the correlations between exposure estimates at birth and at diagnosis (cases) or inclusion (controls) were equal to 0.86 for radon exposure and 0.89 for gamma radiation exposure. A time lag of 24 months was also applied for cumulative exposure in sensitivity analyses.

The cumulative RBM dose was derived from radon and gamma radiation exposures during the intra-uterine period, the first year of life, and the subsequent 14 years of life, using specific conversion factors (see Supplemental Material, “Part 1”). The total RBM dose was calculated as the sum of its radon and gamma radiation components.

### Statistical Analyses

The analyses of the incidence study were conducted using SAS software (version 9.3; SAS Institute Inc., Cary, North Carolina, USA), on the scale of the 36,326 municipalities grouped into categories of exposure to NBR using a Poisson regression model (see Supplemental Material, “Part 2”): Ln[*E*(*O_ka_*)] = Ln(*E_ka_*) + β*_0a_* + β.*D_ka_*, where, Ln[*E*(*O_ka_*)] is the natural logarithm of the expected value of the number of AL cases observed among children of age *a* in the category *k* of exposure to NBR; Ln(*E_ka_*) is the natural logarithm of the corresponding expected number of cases calculated by applying the age-specific incidence rate to the person-years at risk at age *a* in the exposure category *k*; β*_0a_* are age-dependent intercepts; β is the ERR by unit of increase of *D_ka_* and *D_ka_* is the value of the exposure for age *a* and category of exposure *k*.

Bithell’s linear risk score (LRS) test (Bithell 1995) was used to test for the departure of the observations from the null hypothesis of no association with RBM due to radon, gamma radiation, and total NBR exposures against the simple alternative hypothesis that the observations would follow the UNSCEAR 2006 multiplicative ERR model.

The coordinates of the center of the municipality of residence were used for exposure assessment, given their very high correlation with individual exposures to radon or gamma radiation at home for the controls of the Geocap study (*r* = +0.991 for radon and *r* = +0.975 for gamma radiation). To prevent underdispersion in Poisson regression models, the municipalities were classified into 20 exposure categories, with cutoffs chosen to obtain 1/20th of the expected number of cases in each category. Groupings of those categories are specified in the table footnotes in the tabulation of the results. Standardized incidence ratios (SIR) were then calculated as the ratio of the observed (O) to the expected (E) number of cases, and their 95% confidence interval (CI) was calculated using Byar’s approximation (Breslow and Day 1987). For trend analyses, the means of the exposure categories were considered as quantitative variables in Poisson regression models, using the SAS GENMOD (version 9.3) procedure. Analyses were performed using the estimates of external exposure, life-long cumulative exposure, and RBM dose, overall and separately by type of leukemia [acute lymphoid leukemia (ALL), acute myeloid leukemia (AML)], age group (0–4, 5–9, and 10–14 years), and subperiods (1990–1999 and 2000–2009). Analyses by 1-year age group were also conducted to account precisely for possible confounding or effect modification by age.

In additional analyses, the potential for confounding by socioeconomic status was taken into account by stratification on the quintiles of the first component in a principal component analysis of four census variables: median income, percentage of baccalaureate holders, laborers, and unemployed. This socioeconomic indicator was based on the 1999 census and was strongly correlated with the indicators based on the 1990 (*r* = 0.95) and 2006 (*r* = 0.97) censuses. Sensitivity analyses were also conducted by excluding the Paris area, which is the most densely populated area (about 17% of the child population < 2% of the surface area) and has a low exposure to NBR.

The Geocap case–control study was used to control for potential confounding by environmental factors available at the address of residence by exclusion [vicinity of nuclear power plants (Sermage-Faure et al. 2012) or proximity to high-voltage power lines (Sermage-Faure et al. 2013)] or by adjustment [proximity of high traffic roads (Houot et al. 2015)]. The odds ratios (ORs), their 95% CI and two-sided *p*-values were estimated by unconditional logistic regression adjusted for age using the SAS LOGISTIC procedure (version 9.3). Statistical analyses were performed separately for residential exposures to radon or gamma radiation overall, and by type of leukemia (ALL, AML) and age group (0–4, 5–9, and 10–14 years). The exposures were categorized into quintiles based on their distribution in the controls. Logistic linear trend analyses were also conducted.

The statistical significance and power of the tests conducted under different hypotheses of an association between AL incidence and NBR were calculated using a simulation procedure assuming a multiplicative model of the effect of radiation exposures on the incidence rates of AL (see Supplemental Material, “Part 2” and “Part 3”). Realistic alternative hypotheses were *a*) exponential multiplicative models using hypothetical values of 2%, 5%, and 10% for the ERR per mSv, or *b*) the UNSCEAR 2006 multiplicative ERR model, which assumes a lag time of 2 years between radiation exposure and AL risk, and a sharp decrease of the ERR per Sv with attained age (UNSCEAR 2006). Under the alternative hypothesis that the relative risk of AL was an exponential function of cumulative RBM dose, the Poisson regression analysis of the incidence study detected ERRs of 2%, 5%, and 10% by mSv of RBM dose with a power of 97.1%, 100%, and 100%, respectively, for gamma radiation, and with a power of 44.5%, 97.1%, and 100%, respectively, for radon. For total NBR exposure, the power was 100% even for ERRs of 2%. Under the alternative hypothesis that the relative risk of AL fitted the UNSCEAR 2006 multiplicative ERR model, the power of the LRS test to detect an association of observed incidence with NBR exposure was 92.4% for gamma radiation–associated RBM exposure, 44.8% for radon-associated RBM exposure, and 99.4% for total NBR-associated RBM exposures.

The study complied with French regulation on data protection (CNIL 99198; law 78–17 of 6 January 1978) and was exempted from ethical board review. The RNCE has accreditation for cancer registration.

## Results

### Cases

Overall, 9,056 cases of AL (7,434 ALL cases and 1,465 AML cases) were diagnosed over the period 1990–2009 and included in the incidence study, 2,763 of which (2,283 ALL and 418 AML cases) were diagnosed between 2002 and 2007 and included in the Geocap case–control study.

### Exposure to NBR

For the 30,000 Geocap controls, the arithmetic mean for radon exposure estimated at the place of residence was 67.8 Bq/m^3^ (SD = 45.5), the 5th percentile was 24.9 Bq/m^3^, and the 95th percentile was 145.3 Bq/m^3^. The arithmetic mean for gamma radiation exposure was 98.2 nSv/hr (SD = 24.9), the 5th percentile was 70.1 nSv/hr, and the 95th percentile was 148.5 nSv/hr (see Table S1). The corresponding values for the centers of the municipalities of residence were very close to those for the individual addresses. The estimates of the cumulative RBM doses associated with radon and gamma radiation are given in Table S1 for children 5, 10, and 15 years old.

### Incidence Study 1990–2009

There was no association between radon or gamma radiation exposure at diagnosis and the incidence of ALL or AML, and there was no exposure–response linear trend (Table 1).

**Table 1:** Exposure to radon and gamma radiation at the place of residence and risk of acute leukemia in children < 15 years old, RNCE 1990–2009.

Residential exposure	All AL (*n* = 9,056)				ALL (*n* = 7,434)				AML (*n* = 1,465)			
	m	O	E	SIR (95% CI)	m	O	E	SIR (95% CI)	m	O	E	SIR (95% CI)
Radon (Bq/m^3^)^*a*^
12.5–37.1	28.0	1,708	1,815.4	0.94 (0.90, 0.99)	28.0	1,357	1,488.2	0.91 (0.86, 0.96)	28.0	319	295.5	1.08 (0.97, 1.20)
37.2–48.9	43.2	1,864	1,807.1	1.03 (0.99, 1.08)	43.2	1,563	1,483.5	1.05 (1.00, 1.11)	43.2	276	292.4	0.94 (0.84, 1.06)
49.0–62.6	55.3	1,831	1,811.8	1.01 (0.97, 1.06)	55.3	1,496	1,487.7	1.01 (0.96, 1.06)	55.3	305	292.7	1.04 (0.93, 1.17)
62.7–89.7	74.7	1,877	1,812.8	1.04 (0.99, 1.08)	74.7	1,575	1,489.0	1.06 (1.01, 1.11)	74.7	265	292.4	0.91 (0.80, 1.02)
89.8–827.5	137.1	1,776	1,808.9	0.98 (0.94, 1.03)	137.1	1,443	1,485.6	0.97 (0.92, 1.02)	137.1	300	291.9	1.03 (0.92, 1.15)
SIR by 100 Bq/m^3^	—	—	—	1.01 (0.91, 1.12)	—	—	—	1.01 (0.86, 1.19)^*c*^	—	—	—	0.99 (0.81, 1.19)
Gamma radiation (nSv/hr)^*b*^
65.9–80.0	75.2	1,729	1,809.5	0.96 (0.91, 1.00)	75.2	1,420	1,485.7	0.96 (0.91, 1.01)	75.2	283	292.5	0.97 (0.86, 1.09)
80.1–89.6	83.8	1,768	1,811.2	0.98 (0.93, 1.02)	83.8	1,449	1,486.0	0.98 (0.93, 1.03)	83.8	285	293.7	0.97 (0.86, 1.09)
89.7–103.0	96.1	1,894	1,823.5	1.04 (0.99, 1.09)	96.1	1,541	1,497.3	1.03 (0.98, 1.08)	96.1	313	294.6	1.06 (0.95, 1.19)
103.1–123.4	112.9	1,818	1,791.4	1.01 (0.97, 1.06)	112.9	1,496	1,470.5	1.02 (0.97, 1.07)	112.9	297	289.8	1.02 (0.91, 1.15)
123.5–260.8	145.9	1,847	1,820.4	1.01 (0.97, 1.06)	145.9	1,528	1,494.5	1.02 (0.97, 1.07)	145.9	287	294.4	0.97 (0.87, 1.09)
SIR by 10 nSv/hr	—	—	—	1.01 (1.00, 1.02)	—	—	—	1.01 (1.00, 1.02)	—	—	—	1.00 (0.98, 1.02)
Note: —, not applicable; AL, childhood acute leukemia; ALL, acute lymphoid leukemia; AML, acute myeloid leukemia; E, expected number of cases; m, mean weighted by the expected number of cases over 1990–2009; O, observed number of cases; RNCE, National Registry of Childhood Cancers; SIR (95% CI), standardized incidence ratio and its 95% confidence interval (CI) estimated by Poisson regression models for trend analyses with Byar’s approximation for categories based on quintiles. ^***a***^Indoor radon concentration estimated by cokriging. Categories are based on quintiles of the expected number of cases. ^***b***^Gamma radiation estimated by cokriging. Categories are based on quintiles of the expected number of cases. ^***c***^*p*-Value of the test of departure from linearity between 0.01 and 0.05.												

The risk of AL did not increase with cumulative exposures to radon or gamma radiation, overall, by AL type (see Table S2) or by 5-year age groups (see Table S3). Analysis by 1 year age group did not reveal any confounding of modifying effect of age on the association. The results were not changed by incorporating a time lag of 24 months in the analysis (data not shown).

There was no evidence of any association between cumulative RBM dose associated to radon, gamma radiation, or to total NBR and AL incidence, overall, or by 5-year age group (Table 2) or AL type (see Table S4). In all the analyses, the results did not substantially change by the 10-year subperiods (1990–1999 and 2000–2009), or by quintile of socioeconomic indicator, or after exclusion of the Paris area (data not shown). The LRS tests did not reject the hypotheses of no association of AL with radon, gamma, and total NBR exposure against the UNSCEAR 2006 multiplicative ERR model.

**Table 2:** Cumulative red bone marrow dose associated with radon and gamma radiation and risk of acute leukemia in children < 15 years old, RNCE 1990–2009.

Cumulative dose	0–14 years (*n *= 9,056)				0–4 years (*n *= 4,556)				5–9 years (*n *= 2,646)				10–14 years (*n *= 1,854)			
	m	O	E	SIR (95% CI)	m	O	E	SIR (95% CI)	m	O	E	SIR (95% CI)	m	O	E	SIR (95% CI)
Radon (mSv)^*a*^
≤ 2.5	0.9	8,046	8,024.1	1.00 (0.98, 1.02)	0.5	4,517	4,507.8	1.00 (0.97, 1.03)	1.2	2,350	2,351.2	1.00 (0.96, 1.04)	1.7	1,179	1,165.0	1.01 (0.96, 1.07)
2.6–5.0	3.4	819	831.4	0.99 (0.92, 1.06)	2.7	39	48.2	0.81 (0.58, 1.11)	3.3	258	251.3	1.03 (0.91, 1.16)	3.4	522	532.0	0.98 (0.90, 1.07)
5.1–7.5	5.9	127	139.0	0.91 (0.76, 1.09)					5.6	38	43.5	0.87 (0.62, 1.20)	6.1	89	95.5	0.93 (0.75, 1.15)
> 7.5	8.6	64	61.5	1.04 (0.80, 1.33)									8.6	64	61.5	1.04 (0.80, 1.33)
SIR by mSv				1.00 (0.97, 1.02)				1.02 (0.94, 1.10)				1.01 (0.97, 1.05)				0.99 (0.96, 1.02)
Gamma radiation (mSv)^*a*^
≤ 2.5	1.7	1,250	1,271.5	0.98 (0.93, 1.04)	1.7	1,250	1,271.5	0.98 (0.93, 1.04)
2.6–5.0	3.7	2,717	2,711.7	1.00 (0.97, 1.04)	3.6	2,534	2,531.1	1.00 (0.96, 1.04)	4.6	183	180.6	1.01 (0.87, 1.17)
5.1–7.5	6.1	1,825	1,835.4	0.99 (0.95, 1.04)	5.9	683	660.3	1.03 (0.96, 1.12)	6.3	1,142	1,175.2	0.97 (0.92, 1.03)
7.6–10.0	8.7	1,211	1,191.9	1.02 (0.96, 1.08)	8.2	89	93.1	0.96 (0.77, 1.18)	8.6	793	785.1	1.01 (0.94, 1.08)	9.1	329	313.7	1.05 (0.94, 1.17)
10.1–15.0	12.0	1,487	1,467.9	1.01 (0.96, 1.07)					11.7	488	463.7	1.05 (0.96, 1.15)	12.2	999	1,004.2	0.99 (0.93, 1.06)
15.1–20.0	16.9	431	441.0	0.98 (0.89, 1.07)					16.2	40	41.4	0.97 (0.69, 1.32)	17.0	391	399.6	0.98 (0.88, 1.08)
20.1–25.0	21.7	114	117.3	0.97 (0.80, 1.17)									21.7	114	117.3	0.97 (0.80, 1.17)
> 25.0	25.4	21	19.3	1.09 (0.67, 1.66)									25.4	21	19.3	1.09 (0.67, 1.66)
SIR by mSv				1.00 (0.99, 1.01)				1.02 (0.99, 1.05)				1.01 (0.99, 1.03)				1.00 (0.98, 1.01)
Total (mSv)^*a*^
≤ 2.5	1.7	984	1,007.6	0.98 (0.92, 1.04)	1.7	984	1,007.6	0.98 (0.92, 1.04)
2.6–5.0	3.7	2,393	2,404.6	1.00 (0.96, 1.04)	3.7	2,357	2,369.3	1.00 (0.96, 1.04)	4.9	36	35.3	1.02 (0.72, 1.41)
5.1–7.5	6.2	1,752	1,713.1	1.02 (0.98, 1.07)	6.0	966	936.4	1.03 (0.97, 1.10)	6.3	786	776.7	1.01 (0.94, 1.09)
7.6–10.0	8.6	1,177	1,191.0	0.99 (0.93, 1.05)	8.5	218	196.0	1.11 (0.97, 1.27)	8.6	873	911.8	0.96 (0.90, 1.02)	9.4	86	83.2	1.03 (0.83, 1.28)
10.1–15.0	12.2	1,668	1,643.7	1.02 (0.97, 1.07)	11.1	31	46.7	0.66 (0.45, 0.94)	11.9	778	743.3	1.05 (0.97, 1.12)	12.5	859	853.7	1.01 (0.94, 1.08)
15.1–20.0	17.1	699	705.3	0.99 (0.92, 1.07)					16.6	130	136.8	0.95 (0.79, 1.13)	17.2	569	568.6	1.00 (0.92, 1.09)
20.1–25.0	22.0	265	272.3	0.97 (0.86, 1.10)					21.2	43	42.1	1.02 (0.74, 1.38)	22.2	222	230.2	0.96 (0.84, 1.10)
> 25.0	29.1	118	118.3	1.00 (0.83, 1.19)									29.1	118	118.3	1.00 (0.83, 1.19)
SIR by mSv				1.00 (0.99, 1.01)				1.01 (0.99, 1.04)^*b*^				1.01 (0.99, 1.02)				1.00 (0.99, 1.01)
Note: E, expected number of cases; m, mean weighted by the expected number of cases over 1990–2009; O, observed number of cases; RNCE, National Registry of Childhood Cancers; SIR (95% CI), standardized incidence ratio and its 95% confidence interval (CI) estimated by Poisson regression models for trend analyses with Byar’s approximation for categories of exposure. ^***a***^Estimated cumulative red bone marrow dose. The cutoffs are categories of 2.5 mSv up to 10.0 mSv and 5.0 mSv above 10.0 mSv. ^***b***^*p*-Value of the test of departure from linearity between 0.01 and 0.05.																

### Geocap Case–Control Study 2002–2007

The Geocap cases and controls did not differ significantly with respect to their residential exposure to radon and gamma radiation at the residence at diagnosis or inclusion (see Table S5), the cumulative exposures from birth to age at diagnosis or inclusion (results not shown), or the cumulative RBM doses (results not shown). There was no significant association by type of AL (ALL, AML) or age group. The exclusion of the 9 cases and 60 controls living within 50 m of the closest very high voltage power lines (225 kV and 400 kV) and the exclusion of the 14 cases and 80 controls living within 5 km of a nuclear power plant did not modify the results. The results were also similar after adjustment for proximity to roads with heavy traffic.

## Discussion

We did not see evidence of any association between AL incidence rates and exposure to radon or gamma radiation in France in a nationwide incidence study over a 20-year period, using fine scale exposure assessment. None of the metrics used (i.e., radon or gamma radiation exposure, cumulative exposure with the hypothesis of stability since birth, or cumulative RBM dose) was associated with AL overall, ALL or AML, or with any 5-year age group. The large case–control study Geocap, which enabled several additional adjustments, yielded the same results as the incidence study.

In our previous incidence study conducted on the broader scale of the Département (i.e., a French administrative unit between a municipality and region) for the period 1990–2001, we observed no increase in the risk of AL with exposure to gamma radiation, but we showed a moderate and significant association between residential radon exposure and the incidence of AML in children: There was a 20% excess risk in the Départements with the highest radon exposure (on average, there was a difference in exposure of 100 Bq/m^3^ between the highest and lowest quintile taken as reference quintile) (Evrard et al. 2006). In the present study, for the period 1990–2010, we found no increase in AML risk with any of the radon exposure metrics under study, when local exposure was estimated more precisely and on a much finer scale than in the previous study.

A Danish record-based case–control study, which included 2,400 cases of AL for the period 1968–1994 (Raaschou-Nielsen et al. 2008) showed a clear association with lifelong exposure to domestic radon, based on residential history. In contrast to our previous study, the association was specific to ALL and not to AML. A Swiss cohort, which included 149 AL cases for the period 2000–2008 (Hauri et al. 2013), did not show any association with radon exposure. Neither did a Norwegian cohort study, which included 437 cases of AL for the period 1967–2009 (Del Risco Kollerud et al. 2014). A nationwide record-based case-control study in the UK, which included the 9,058 cases of AL registered over 1980–2006 and a birth-matched random sample of 11,912 controls, evidenced an association with cumulative gamma radiation exposure (ERR = 9%/mSv; 95% CI: 2%, 17%) but not with radon exposure (Kendall et al. 2013). A Swiss record-based cohort study, which included all the Swiss children < 16 years old in the 1990 and 2000 Swiss National Censuses, identified 530 observed cases of AL in the follow-up period 1990 to 2008 and showed an increase in risk with cumulative gamma radiation exposure (ERR = 4%/mSv; 95% CI: 0%, 8%) (Spycher et al. 2015).

In the present study, the estimates of exposures for the municipalities of residence appeared to be as precise as the estimates at the individual addresses that were available for all the cases and for the representative controls sampled for the Geocap study. With the model we used, residence location explained 32% (*r* = 0.57) of the total variability of radon between residences and 65% (*r* = 0.81) of the total variability of terrestrial gamma radiation between residences. Using model-based average estimates as surrogates of individual measurements has probably not induced any bias in the estimates of the associations, given the degree of local aggregation of exposure data [Berkson type of error (Steenland et al. 2000)]. Indeed, individual exposures or log-exposures were likely to be adequately modeled as the sum of average local exposure and independent unmeasured individual increments. The misclassification is consequently expected to produce a loss of statistical power related to a shrinkage of the exposure range, but no bias in the estimate of the association.

Regarding radon exposure, the quality of our exposure models seems comparable to that of the studies in Denmark, the United Kingdom, Norway, and Switzerland (Del Risco Kollerud et al. 2014; Hauri et al. 2013; Kendall et al. 2013; Raaschou-Nielsen et al. 2008; Spycher et al. 2015). Thus, quality is unlikely to constitute the explanation for the divergent results. In the Danish study (Andersen et al. 2007), residential exposure to radon was predicted by a model including geological data and housing characteristics, which explained 40% of the variability of nearly 3,000 domestic radon measurements. The study had access to information on individual housing characteristics from the data of the Danish Building and Dwelling Register. In the Swiss study (Hauri et al. 2013), the estimated radon exposure was also predicted by a model including geological data and housing characteristics that explained 20% of the variance of radon concentration. In the Norwegian study (Del Risco Kollerud et al. 2014), radon exposure was estimated as the geometric mean of indoor radon measurements (for a total set of 41,515 measurements) using a buffer model with different radii. In that study, the intraclass correlation coefficients between estimates from the buffer model and indoor radon measurements varied from 0.63 to 0.68 depending on the number of measurements in the buffer (Kollerud et al. 2014). The UK study (Kendall et al. 2013) estimated residential radon concentrations using the average exposure of the County District calculated from 2,283 measurements generated by a national campaign (Wrixon et al. 1988), and a predictive map of radon exposure based on the results of about 400,000 measurements grouped by 1 × 1 km^2^ square and geological units (Appleton and Miles 2010; Miles and Appleton 2005). Between 34% and 40% of the variance in the radon concentration was explained, depending on the geology (Appleton and Miles 2010).

With respect to gamma radiation, the study by Kendall et al. (2013) in the United Kingdom used average estimates by County District [average population: 100,000 (Kendall et al. 2006)] based on a set of 2,283 measurements. It should be noted that they recently proposed a new and improved approach using a combination of geological codes and inverse power of distance weights of the dose rates at neighboring points (Kendall et al. 2016). In the Swiss study (Spycher et al. 2015), the dose rate map relied on a database with a density of about 1 point per 25 km^2^. The data used in our study (Warnery et al. 2015) combined a learning sample of 97,795 *in situ* gamma radiation measurements in 17,404 locations, a map of the geogenic uranium potential, and geostatistical modeling. Using this method (Warnery et al. 2015), 65% of the total variability of telluric gamma radiation measurements could be explained by the spatial coordinates of the home, which is of the same order of magnitude as in the United Kingdom and Swiss studies (Kendall et al. 2013; Spycher et al. 2015).

France has a wide range of radon and gamma radiation exposures. In Denmark, where an association between ALL and radon was reported, exposure to radon ranged from 4 to 254 Bq/m^3^ with a mean of 48 Bq/m^3^, which is not particularly high compared with other European countries. In France, estimated exposure to radon ranged from 12.5 to 819.2 Bq/m^3^ among the Geocap controls, with an arithmetic mean of 67.2 Bq/m^3^. In the United Kingdom, the range was 1.2 to 692 Bq/m^3^ with a lower arithmetic mean of 21.3 Bq/m^3^. In Switzerland, the range was 0.7 to 490 Bq/m^3^ with an arithmetic mean of 86 Bq/m^3^. In Norway, the mean radon concentration for the whole cohort was 91 Bq/m^3^ and the median was 74 Bq/m^3^.

Differences in gamma radiation ranges between the United Kingdom, Switzerland, and France also appear too small to explain the divergence of the published results. In France, estimated gamma exposure by municipality ranged from 65.9 nSv/hr to 260.8 nSv/hr, with an arithmetic mean of 102.6 nSv among the 30,000 Geocap controls. This is of the same order of magnitude as in the UK study with a mean for controls of 94.7 (SD 15.6; range 38.1 to 159.7) nGy/hr. Gamma exposure by county district ranged from 38 nGy/hr to 169 nGy/hr with an arithmetic mean of 93.6 nGy/hr. In the Swiss study, gamma radiation exposure ranged from 55 nSv/hr to 383 nSv/hr with an arithmetic mean of 109 nSv/hr.

In our study, cumulative exposure was calculated from the residence at diagnosis with the hypothesis of constant exposure to NBR from birth until diagnosis. In the Danish and Norwegian studies, cumulative exposure was based on the residential history, but in the UK study, cumulative exposure was extrapolated from the residences at birth. In the Swiss study, exposure from birth to diagnosis was extrapolated from exposure at census. To evaluate the impact of the misclassification of cumulative exposure, we took advantage of the residential history recorded in the interview-based nationwide case–control study ESCALE, which we conducted in 2003–2004 (Rudant et al. 2007). In that study, 34% of the children had moved to another municipality since birth. Using the same model as in the main analyses, the estimates of residential exposures at birth and at the time of the study were closely correlated (0.86 for radon and 0.89 for gamma radiation, overall). Similarly, Kendall et al. (2015) observed that the estimated dose at the diagnosis address was strongly correlated with that at the birth address in their UK case-control study, in which children were included at birth. If there is a true relationship between AL and cumulative NBR dose, it should be more underestimated by misclassification in the oldest children than in the youngest. However, this is not supported by our results stratified by age group.

Accounting for contextual urbanization and socioeconomic indicators defined on the scale of the municipalities had no influence on the results of the incidence study. In addition, adjustment for the individual environmental exposures associated with AL in our previous analyses of the Geocap study did not modify the results on radiation, suggesting that these factors have little or no confounding effect on the association with NBR. However, it remains possible that unmeasured confounders have masked an association. Our study does not address diagnostic radiology and low-dose radiation other than NBR but is unlikely to have biased our estimates because their distribution is not expected to depend on NBR exposures. Our conclusions are therefore limited to NBR and cannot be generalized to all low-dose radiation exposures.

Our study has many strengths including its size (9,056 cases), the completeness of AL cases registration (three sources by case on average), the contrasts in average local exposures to NBR over the country, the precision of the exposure estimates and the use of two study designs. The study had sufficient power to reveal an association between gamma radiation exposure and AL under the plausible hypotheses that the association fitted the UNSCEAR multiplicative ERR model (UNSCEAR 2006) or a log-linear model with cumulative exposure producing ERR of 2% to 10% per mSv of RBM dose.

In contrast to the British and Swiss studies, our study is based on exposures estimated at the time of diagnosis and this may be a reason for our discrepant results on gamma radiation. Exposures at birth may be more relevant, and we are now trying to collect data for that period. We do not expect marked differences since the exposures to NBR during the two periods were available and strongly correlated in our ESCALE study, but careful testing is nevertheless warranted.

## Conclusions

Our findings do not support the hypothesis that residential exposure to NBR increases the risk of AL, despite the study’s large size, fine scale of exposure estimates and wide range of exposures over France. However, our results at the time of diagnosis do not rule out a slight association with gamma radiation at the time of birth, which would be more in line with the recent findings in the United Kingdom and Switzerland. Future studies should try to account for the complete residential history, or at least its two extremes, in order to enhance the evaluation of the AL risk associated with NBR in children.

## Supplemental Material

(372 KB) PDF
