# Supplementary material for: Residential Exposure to Natural Background Radiation and Risk of Childhood Acute Leukemia in France, 1990–2009
Source: Environ Health Perspect. 2016 Aug 2;125(4):714–20. doi: 10.1289/EHP296 (PMC5381982; doi:10.1289/EHP296)
Supplement: (372 KB) PDF [file EHP296.s001.acco.pdf]

Environ Health Perspect
DOI: 10.1289/EHP296

Note to readers with disabilities: *EHP* strives to ensure that all journal content is accessible to all readers. However, some figures and Supplemental Material published in *EHP* articles may not conform to 508 standards due to the complexity of the information being presented. If you need assistance accessing journal content, please contact ehp508@niehs.nih.gov. Our staff will work with you to assess and meet your accessibility needs within 3 working days.

Supplemental Material

Residential Exposure to Natural Background Radiation and Risk of Childhood Acute Leukemia in France, 1990 - 2009

Claire Demoury, Fabienne Marquant, Géraldine Ielsch, Stéphanie Goujon, Christophe Debayle, Laure Faure, Astrid Coste, Olivier Laurent, Jérôme Guillevic, Dominique Laurier, Denis Hémon, and Jacqueline Clavel

Table of Contents

Part 1. Exposure metrics

Part 2. Statistical modeling and analysis of observations using a log-linear multiplicative model, estimation of statistical power

Part 3. Testing for the existence of an association between AL and RBM dose using the UNSCEAR 2006 ERR model

References

Table S1. Distribution of exposure to natural background radiation and estimated dose to red bone marrow in controls, Geocap study 2002-2007

Table S2. Cumulative exposure to radon and gamma radiation, and risk of acute leukemia, ALL and AML in children under 15 years old, RNCE 1990-2009

Table S3. Cumulative exposure to radon and gamma radiation, and risk of acute leukemia in children under 15 years old, by 5-year age group, RNCE 1990-2009

Table S4. Cumulative red bone marrow dose associated to radon and gamma radiation, and risk of ALL and AML in children under 15 years old, RNCE 1990-2009

Table S5. Exposure to radon and gamma radiation, and risk of acute leukemia in children under 15 years old, Geocap study 2002-2007

Part 1. Exposure metrics

Exposure

The exposures to radon $d_i(Rn)$ in Bq/m^3 and gamma radiation $d_i(\gamma)$ in nSv/h (telluric + cosmic) were directly available for each of the 36,326 French mainland municipalities.

<u>*Exposure to radon*</u>

During a national campaign conducted by the Institute for Radiation Radiological Protection and Nuclear Safety (IRSN) indoor radon activity per cubic meter (Bq/m^3) was measured in 10,843 houses in the main room, living room or bedroom, over two months, using an open Kodalpha LR115 passive track-etch detector (Billon et al. 2005). Seasonal variations in the indoor radon concentrations were accounted for by applying a correction factor as per the method described by Baysson et al. (2003) (Baysson et al. 2003). The complete address was available for 8,136 measurements that were geocoded at the address point. The other measurements were geocoded at the municipality center point.

In order to identify radon-prone areas in France, the IRSN developed a harmonized methodology to derive a single map of the geogenic radon potential (GRP) (Demoury et al. 2013; Ielsch et al. 2010; Ielsch et al. 2014). This approach consists in determining the capacity of the geological units to produce radon and facilitate its transfer to the atmosphere. The approach is first based on a classification of the geological units by their uranium content, in order to create a radon source potential map. The initial map is then improved by taking into account the main additional parameters, which control the preferential pathways of radon through the ground and may increase the radon potential. The final map results in the categorization of France in five classes: from low to high GRP with accurate geographic resolution.

The cokriging estimates were based on the measurements made during the national campaign and the GRP was used as a regionalized variable in the cokriging (IRSN Report 2012; Ielsch et al. 2015).

Exposure to terrestrial gamma radiation

In 2011-2012, 97,595 measurements of gamma rays were collected by the IRSN in 17,404 dentist surgeries and veterinary clinics all over France, using Radio Photo Luminescent dosimeters. Only the measurements made with dosimeters not exposed to anthropic sources were used for the model. After subtracting the cosmic ray component determined using the UNSCEAR altitude-based formula (2000), the arithmetic mean of the time-series measurements, weighted by the time-exposure of the dosimeters, was used as the value of the exposure of each location to indoor terrestrial gamma radiation (Warnery et al. 2015).

In 2010, the IRSN classified the French geological formations, characterizing their uranium potential on the bases of geology, petrology and local determination of the uranium content of rock (Ielsch et al., in press). This information was geo-referenced on a map on the scale 1:1,000,000. The geological uranium potential was classified as 5 qualitative categories. Since telluric gamma radiation is mostly derived from the progenies of the (238) uranium series present in rocks, this information, which is exhaustive throughout France, was incorporated in the estimates of telluric gamma radiation dose rates.

The gamma radiation exposures of the French municipalities were estimated as the average gamma radiation dose rate of the 1 x 1 km squares whose centers lay within the perimeter of the municipality. Similarly the gamma exposure of each Geocap subject was the gamma dose rate of the 1 x 1 km square in which the residence was geolocated.

Cumulative exposure

Assuming that the exposures were constant from birth till age *a* at diagnosis (or inclusion for the controls), the cumulative exposures to radon $CE_i(Rn)$ and gamma radiation $CE_i(\gamma)$ in municipalities *i* are given by:

$CE_i(Rn) = a \times d_i(Rn)$ in Bq/m^3.year

and $CE_i(\gamma) = a \times d_i(\gamma) \times (24 \times 365.25/1{,}000{,}000)$ in mSv.year.

where di(Rn) in Bq/m^3 and di(γ) in nSv/h are the exposures to radon and gamma radiation (telluric + cosmic) respectively, in each of the 36,326 French continental municipalities i.

Cumulative red bone marrow (RBM) dose

Using conversion coefficients C_u of exposures at age *u*, the RBM dose accumulated at age *a* was calculated as:

$RBM_i(Rn) = d_i(Rn) \times (\sum_{u=0}^{a} C_u(Rn))$ and

$RBM_i(\gamma) = d_i(\gamma) \times (24 \times 365.25/1{,}000{,}000) \times (\sum_{u=0}^{a} C_u(\gamma))$,

where di(Rn) in Bq/m^3 and di(γ) in nSv/h are the exposures to radon and gamma radiation (telluric + cosmic) respectively, in each of the 36,326 French continental municipalities i and C_u are the exposure-dose conversion coefficients at age u. For radon and its decay products, C_u considered a radiation biological effectiveness factor of 20 applied to alpha emitters. Coefficients C_u took the values given in the table below.

Table: Conversion coefficients C_u of exposure to RBM dose by age at exposure u

Age at exposure *u*	Radon [a] (Bq/m^3)	Gamma[b] (mSv/year)
Intra-uterine exposure (mSv)	0.00025	0.675
0 to 1 year of age (mSv/year)	0.00155	1.240
1 to 15 years of age (mSv/year)	0.00315	1.120

[a] Coefficients based on (Kendall and Smith 2002, 2005); [b] Coefficients based on (Petoussi et al. 1991)

In the UNSCEAR ERR 2006 model, the RBM dose was calculated assuming no intra-uterine component of exposure and a two-year time lag for the increase in risk following radiation exposure, i.e. cumulative from birth until age a-2 (UNSCEAR 2006).

Part 2. Statistical modeling and analysis of observations using a log-linear multiplicative model, estimation of statistical power

Statistical modeling

Under the assumption of a log-linear multiplicative model, the 1990-2009 incidence study and the Geocap case-control study (2002-2007) were analyzed using a log-linear Poisson model and a linear-logistic model, respectively. The modeling is briefly presented here for the incidence study:

The multiplicative-exponential model is written:

$$h(D_{ia},a)=h(0,a) \times \exp(ß \times D_{ia}) \qquad (1),$$

where:

- D_{ia} is the metric for dose of radiation (exposure, cumulative exposure, cumulative RBM dose) at age a for children living in municipality i
- $h(D_{ia}, a)$ is the hazard rate for children of age a exposed to D_{ia},
- $h(0,a)$ is the hazard rate for children of age a in the absence of any exposure to natural background radiation (NBR),
- ß is the excess relative risk (ERR) by unit of exposure of D_{ia}.

,

which gives, by multiplying both sides of equation (1) by the adequate number of person-years at risk and summing over the 20 years of the study, and after a few simple algebraic procedures:

$$Ln(E(O_{ia}))=Ln(E_{ia})+\underline{ß_{0a}}+ ß \times D_{ia} \quad (2)$$

where:

- $E(O_{ia})$ is the expected value of the observed number of cases O_{ia},
- E_{ia} is the expected number of cases derived from the number of person-years at risk among children of age a in municipality i and from the observed French age-specific AL incidence rates
- $ß_{0a}$ are age dependent intercepts.

Due to the very large number of cells (36,326 municipalities by 15 one-year classes of age = 543,540 cells), adjusting a Poisson regression model to equation (2) led to an under-dispersion of the observed counts O_{ia}. For this reason, the municipalities were grouped together by level of exposure into 20 categories (k = 1 to 20) with equal expected numbers of cases. The number of cells was thus reduced to 300 (20 categories of exposure by 15 categories of age) and equation (2) was re-written as equation (3), which prevented over-dispersion and under-dispersion:

$$Ln(E(O_{ka}))=Ln(E_{ka})+\underline{ß_{0a}}+ ß \times D_{ka} \quad (3)$$

Model (3) was used overall (age < 15 years), or for each of the 15 one-year classes, or 3 five–year classes of age.

Using model (3), it was possible to estimate ERR per Sv ß and to test the null hypothesis H_0 of no association between AL and NBR (ß = 0) against the composite alternative hypothesis H_1 that AL was associated with RBM dose associated to radon, gamma radiation or both exposures considered together (ß ≠ 0).

Estimating the power of the statistical tests conducted

Simulation procedure

Choosing a specific non-zero plausible alternative value ß* for the unknown ERR, the power of the test of H_0 against H_1 was estimated using the following simulation procedure:

- from the value of ß*, the expectation of the observed number of cases $E(O_{ka})$ was derived from equation (3),
- then, for each of the 15 one-year classes of age *a*, the observed counts O_{ka} were randomly generated by allocating the O_{+a} total observed number of cases of age *a* to the 20 categories of municipalities, using a multinomial distribution with probability proportional to its expected number of cases $E(O_{ka})$ under H_1.
- finally, equation (3) was fitted to the numbers of observed (O_{ka}) and expected (E_{ka}) cases in the 300 observation cells. The resulting estimate of ß was compared to the null value ß = 0 from which it was significantly different or not in a two-sided test of H_0 against H_1 at the level $\alpha = 5\%$, based on the simulated statistical distribution of 10,000 independent estimates of ß under the hypothesis H_0.

Choosing plausible values of ERR ß for power calculations*

Recently, (Wakeford 2013) concluded from his review of the available evidence on the quantitative association between exposure to ionizing radiation and the risk of childhood leukemia (AL), that an ERR of 50 per Sv was *"broadly applicable to low dose, low-dose rate exposure circumstances"*. In a recent UK case-control study, Kendall et al. (2013) (Kendall et al. 2013) observed an ERR of + 12% (95%CI: + 3%, + 22%) for an increase of 1 mSv in gamma radiation-associated RBM dose and an ERR of + 9% (95%CI, +2% +17%) for an increase of 1 mGy in gamma radiation cumulative exposure (corresponding to ERR values of

120 and 90 per Sv, respectively). In a recent Swiss cohort study, Spycher et al. (2015) (Spycher et al. 2015) observed a relative risk of 1.046 (95% CI 0.999, 1.096) for a 1 mSv increase in cumulative exposure to gamma radiation (corresponding to an ERR of 46 per Sv).

Considering these observations, ERR per mSv of RBM dose of +2%, +5%, +10% (corresponding respectively to ERRs of 20, 50 and 100 per Sv) were considered as plausible alternatives to the absence of association between NBR exposure and AL risk. The power of the present investigation to detect an association between AL risk and NBR exposure was calculated using the potential ERR per mSv values.

Part 3. Testing for the existence of an association between AL and RBM dose using the UNSCEAR 2006 ERR model.

The model proposed by the UNSCEAR in its 2006 report for estimation of the risk of leukemia is a linear-quadratic multiplicative ERR model, considering a minimum lag time of 2 years between exposure and risk of leukemia (UNSCEAR 2006). The relative risk at age *a* for exposure $RR(D_{ia},a)$ writes as follows:

$$RR(D_{ia},a) = 1 + \alpha \times D_{ia} \times (1 + \beta/\alpha \times D_{ia}) \times (\exp(\kappa_1 \times Ln(a))), \qquad (4)$$

where:

- D_{ia} is the RBM dose (Sv) accumulated up to age *a*-2,

- $\alpha = 864.552$, $\beta/\alpha = 1.18092$ and $\kappa_1 = -1.647$ were estimated from cohorts exposed to ionizing radiation, including the Life Span Study of Hiroshima and Nagasaki A-bomb survivors.

As this model is presently considered as a reference for the estimation of AL risks associated with ionizing radiation, the observations made in the present study were also used to test the

null hypothesis H_0 of no association between AL and NBR against the simple alternative hypothesis H_1 that the observations would fit the UNSCEAR 2006 multiplicative ERR model.

In that case, the alternative hypothesis being a simple one, the most powerful test T of H_0 against H_1 is based on the Likelihood Ratio Score (LRS) statistic of (Bithell 1995):

$$T = \sum_{ka} O_{ka} . \operatorname{Ln}[RR(D_{ka}, a) / RR(\bullet_a)]$$

where O_{ka} is the number of cases observed in the cells (k,a), $RR(D_{ka}, a)$ is the value of the relative risk given by equation (4), and $RR(\bullet a)$ is for age *a*, the weighted average value of $RR(D_{ka},a)$ with weights E_{ka}.

The power of the test of H_0 against H_1 may be estimated using the simulation procedure described above in Part 2 when no "plausible" values has to be chosen for unknown parameters since the alternative hypothesis is a simple one with specified values for its parameters, and the relative risk of AL associated with an exposure D_{ka} accumulated until age *a* is now given by equation (4).

References

Baysson H, Billon S, Laurier D, Rogel A, Tirmarche M. 2003. Seasonal correction factors for estimating radon exposure in dwellings in France. Radiat Prot Dosimetry 104:245-252.

Billon S, Morin A, Caer S, Baysson H, Gambard JP, Backe JC, et al. 2005. French population exposure to radon, terrestrial gamma and cosmic rays. Radiat Prot Dosimetry 113:314-320.

Bithell JF. 1995. The choice of test for detecting raised disease risk near a point source. Stat Med 14:2309-2322.

Demoury C, Ielsch G, Hemon D, Laurent O, Laurier D, Clavel J, et al. 2013. A statistical evaluation of the influence of housing characteristics and geogenic radon potential on indoor radon concentrations in France. J Environ Radioact 126:216-225.

Ielsch G, Cushing ME, Combes P, Cuney M. 2010. Mapping of the geogenic radon potential in France to improve radon risk management: Methodology and first application to region Bourgogne. J Environ Radioact 101:813-820.

Ielsch G, Cuney M., Rossi F., Buscail F., Girard A., Leon A., Cushing M.E., Guillevic J., 2014. Geogenic radon potential mapping in France and French Overseas Territories. The 9th International Symposium on the Natural Radiation Environment (NRE-IX), 22-26 September, 2014, Hirosaki, Japan.

Ielsch G., Warnery E., Pouchol C., Lajaunie C., Cale E., Wackernagel H. 2015. Mapping of indoor radon and terrestrial gamma radiation levels in France: geostatistical modeling. Radon in the Environment, 2nd International Conference, Krakow, Poland, May 25-29, 2015.

Ielsch G, Cuney M., Buscail F., Rossi F., Leon A., Cushing M.E. In press, 2016. Estimation and mapping of uranium content of geological units in France. J Environ Radioact. doi:10.1016/j.jenvrad.2016.05.022.

IRSN Report. 2012. PRP-DGE 2012-018. Analyse spatiale et cartographie du radon sur le territoire métropolitain par l'utilisation de méthodes géostatistiques [in French]. Institut de Radioprotection et de Sûreté Nucléaire, France.

Kendall GM, Smith TJ. 2002. Doses to organs and tissues from radon and its decay products. J Radiol Prot 22:389-406.

Kendall GM, Smith TJ. 2005. Doses from radon and its decay products to children. J Radiol Prot 25:241-256.

Kendall GM, Little MP, Wakeford R, Bunch KJ, Miles JC, Vincent TJ, et al. 2013. A record-based case-control study of natural background radiation and the incidence of childhood leukaemia and other cancers in Great Britain during 1980-2006. Leukemia 27:3-9.

Petoussi N, Jacob P, Zankl M, Saito K. 1991. Organ doses for foetuses, babies, children and adults from environmental gamma rays. Radiat Prot Dosimetry 37:31-41.

Spycher BD, Lupatsch JE, Zwahlen M, Roosli M, Niggli F, Grotzer MA, et al. 2015. Background ionizing radiation and the risk of childhood cancer: A census-based nationwide cohort study. Environ Health Perspect 123:622-628.

UNSCEAR (United Nations Scientific Committee on the Effects of Atomic Radiation). UNSCEAR 2000 Report to the general assembly, with scientific annexes. Sources and effects of ionizing radiation, Vol. I. United Nations, New York.

UNSCEAR (United Nations Scientific Committee on the Effects of Atomic Radiation). UNSCEAR 2006 Report to the general assembly, with scientific annexes. Effects of ionizing radiation, Vol. I. United Nations, New York.

Wakeford R. 2013. The risk of childhood leukaemia following exposure to ionising radiation--a review. J Radiol Prot 33:1-25.

Warnery E, Ielsch G, Lajaunie C, Cale E, Wackernagel H, Debayle C, et al. 2015. Indoor terrestrial gamma dose rate mapping in France: A case study using two different geostatistical models. J Environ Radioact 139:140-148.

Table S1. Distribution of exposure to natural background radiation and estimated dose to red bone marrow in controls, Geocap study 2002-2007

	Mean	SD	Min	p5%	p25%	p50%	p75%	p95%	Max
**Radon**									
Radon exposure (Bq/m^3) at the residence	67.8	45.5	12.8	24.9	40.6	55.6	82.0	145.3	801.3
Radon exposure (Bq/m^3) at the town hall of the municipality	67.2	45.7	12.5	21.8	40.7	55.2	81.6	145.6	819.2
RBM dose associated to radon exposure[a] (mSv)									
At 5 years old	1.0	0.66	0.2	0.3	0.6	0.8	1.2	2.1	11.8
At 10 years old	2.0	1.38	0.4	0.7	1.2	1.7	2.5	4.4	24.7
At 15 years old	3.1	2.10	0.6	1.0	1.9	2.5	3.7	6.7	37.6
**Gamma radiation**									
Gamma radiation exposure (nSv/h) at the residence[b]	98.2	24.9	62.4	70.1	78.1	91.8	112.5	148.5	254.7
Mean value of gamma radiation (nSv/h) in the municipality	102.6	26.3	65.9	73.1	81.1	95.8	117.6	156.9	260.8
RBM dose associated to gamma radiation [c] (mSv)									
At 5 years old	5.8	1.47	3.7	4.1	4.5	5.4	6.6	8.8	14.6
At 10 years old	10.8	2.76	6.9	7.7	8.5	10.1	12.4	16.5	27.4
At 15 years old	15.8	4.05	10.2	11.3	12.5	14.8	18.1	24.2	40.2

Mean: arithmetic mean; SD: standard deviation; Min: minimum observed average local exposure; p: percentile; Max: maximum observed average local exposure; RBM: red bone marrow

[a] Calculated assuming exposure constant from the intra-uterine period until age reached and no lag time, using the conversion coefficients of reference (Kendall and Smith 2002, 2005)

Estimated RBM dose (mSv) associated to radon exposure until age a = [radon exposure (Bq/m^3)] x [0.00025 + 0.00155 + (age - 1) x 0.00315] when age $\geq$ 1

Estimated RBM dose (mSv) associated to radon exposure until age a = [radon exposure (Bq/m^3)] x [0.00025 + 0.00155] when age < 1

[b] 270 missing values

[c] Calculated assuming constant exposure from the intra-uterine period until age reached and no lag time, using the conversion coefficients of reference (Petoussi et al. 1991)

Estimated RBM dose (in mSv) associated to gamma exposure until age a = [Gamma dose-rate (nSv/h)] x [24 x 365.25/1,000,000] x [0.675 + 1.24 + (age-1) x 1.12] when age $\geq$ 1.

Estimated RBM dose (mSv) associated to gamma exposure until age a = [Gamma dose-rate (nSv/h)] x [24 x 365.25/1,000,000] x [0.675 + 1.24] when age < 1.

Table S2. Cumulative exposure to radon and gamma radiation, and risk of acute leukemia, ALL and AML in children under 15 years old, RNCE 1990-2009

Type of leukemia	All AL (N=9,056)				ALL (N=7,434)				AML (N=1,465)			
	Mean	O	E	SIR (95% CI)	Mean	O	E	SIR (95% CI)	Mean	O	E	SIR (95% CI)
**Radon (Bq/m^3 .year)** [a]												
[0-118.4[	68.4	1,802	1,805.6	1.00 (0.95, 1.04)	74.0	1,302	1,298.8	1.00 (0.95, 1.06)	54.2	458	463.8	0.99 (0.90, 1.08)
]118.4;215.3]	166.9	1,816	1,827.1	0.99 (0.95, 1.04)	167.1	1,624	1,616.4	1.00 (0.96, 1.05)	165.4	172	189.3	0.91 (0.78, 1.06)
]215.3;358.8]	286.2	1,748	1,792.6	0.98 (0.93, 1.02)	285.8	1,493	1,565.8	0.95 (0.91, 1.00)	289.1	225	200.8	1.12 (0.98, 1.28)
]358.8;618.1]	479.0	1,936	1,830.0	1.06 (1.01, 1.11)	478.0	1,605	1,527.3	1.05 (1.00, 1.10)	484.6	303	272.1	1.11 (0.99, 1.25)
]618.1;895.7]	740.0	872	894.1	0.98 (0.91, 1.04)	739.2	711	712.2	1.00 (0.93, 1.07)	743.3	142	164.3	0.86 (0.73, 1.02)
]895.7;1189.0]	1,010.9	400	401.8	1.00 (0.90, 1.10)	1,008.4	322	319.8	1.01 (0.90, 1.12)	1,020.6	68	74.0	0.92 (0.71, 1.16)
> 1189.0	1,698.1	482	504.8	0.95 (0.87, 1.04)	1,686.5	377	393.7	0.96 (0.86, 1.06)	1,740.3	97	100.6	0.96 (0.78, 1.18)
SIR by 1000 Bq/m^3 .year				0.99 (0.93, 1.06)				1.01 (0.94, 1.09) [b]				0.89 (0.76, 1.04)
**Gamma radiation (mSv.year)** [a]												
≤ 2.1	1.2	1,823	1,834.9	0.99 (0.95, 1.04)	1.4	1,277	1,288.1	0.99 (0.94, 1.05)	1.0	503	502.9	1.00 (0.91, 1.09)
]2.1;3.5]	2.8	1,776	1,797.9	0.99 (0.94, 1.03)	2.8	1,615	1,633.0	0.99 (0.94, 1.04)	2.7	142	146.2	0.97 (0.82, 1.14)
]3.5;5.4]	4.4	1,824	1,803.6	1.01 (0.97, 1.06)	4.4	1,634	1,613.5	1.01 (0.96, 1.06)	4.5	164	165.2	0.99 (0.85, 1.16)
]5.4;8.8]	7.0	1,818	1,818.6	1.00 (0.95, 1.05)	7.0	1,514	1,521.1	1.00 (0.95, 1.05)	7.2	274	267.1	1.03 (0.91, 1.16)
]8.8;11.2]	9.9	928	891.3	1.04 (0.98, 1.11)	9.9	713	691.4	1.03 (0.96, 1.11)	9.9	191	181.7	1.05 (0.91, 1.21)
]11.2;13.4]	12.1	435	447.7	0.97 (0.88, 1.07)	12.1	331	341.7	0.97 (0.87, 1.08)	12.1	96	95.9	1.00 (0.81, 1.22)
> 13.4	16.0	452	461.9	0.98 (0.89, 1.07)	15.9	350	345.2	1.01 (0.91, 1.13)	16.0	95	106.0	0.90 (0.73, 1.10)
SIR by 1 mSv.year				1.00 (0.99, 1.01)				1.01 (0.99, 1.02)				0.99 (0.96, 1.02)

RNCE: National Registry of Childhood Cancers; AL: childhood acute leukemia; ALL: acute lymphoid leukemia; AML: acute myeloid leukemia Mean: mean weighted by the expected number of cases over 1990-2009; O: observed number of cases; E: expected number of cases; SIR (95% CI): standardized incidence ratio and its 95% confidence interval estimated by Poisson regression models for trend analyses, with Byar's approximation otherwise.

[a] Estimated cumulative exposure. The cutoffs of the categories of exposure are based on the distribution of the expected number of cases, at the following percentiles p20%, p40%, p60%, p80%, p90%, p95%.

[b] p value of the test of departure from linearity between 0.01 and 0.05

Table S3. Cumulative exposure to radon and gamma radiation, and risk of acute leukemia in children under 15 years old, by 5-year age group, RNCE 1990-2009

Age	0-4 y (N=4,556)				5-9 y (N=2,646)				10-14 y (N=1,854)			
	Mean	O	E	SIR (95% CI)	Mean	O	E	SIR (95% CI)	Mean	O	E	SIR (95% CI)
**Radon (Bq/m^3 .year)** [a]												
[0-118.4[	67.6	1,758	1,770.8	0.99 (0.95, 1.04)	108.1	44	34.8	1.26 (0.92, 1.70)				
]118.4;215.3]	164.8	1,502	1,493.9	1.01 (0.95, 1.06)	175.0	288	317.3	0.91 (0.81, 1.02)	206.3	26	15.9	1.64 (1.07, 2.40)
]215.3;358.8]	279.7	822	828.1	0.99 (0.93, 1.06)	292.0	784	812.4	0.97 (0.90, 1.03)	290.8	142	152.2	0.93 (0.79, 1.10)
]358.8;618.1]	465.9	375	357.7	1.05 (0.94, 1.16)	471.8	958	890.0	1.08 (1.01, 1.15)	498.1	603	582.3	1.04 (0.95, 1.12)
]618.1;895.7]	730.7	60	57.3	1.05 (0.80, 1.35)	729.1	310	326.4	0.95 (0.85, 1.06)	748.0	502	510.4	0.98 (0.90, 1.07)
]895.7;1189.0]	939.7	39	48.2	0.81 (0.58, 1.11)	996.6	100	107.4	0.93 (0.76, 1.13)	1,031.0	261	246.3	1.06 (0.93, 1.20)
> 1189.0					1,476.1	162	157.8	1.03 (0.87, 1.20)	1,799.1	320	347.0	0.92 (0.82, 1.03)
SIR by 1000 Bq/m^3 .year				1.06 (0.85, 1.32)				1.02 (0.91, 1.15)				0.96 (0.88, 1.05)
**Gamma radiation (mSv.year)** [a]												
≤ 2.1	1.2	1,823	1,834.9	0.99 (0.95, 1.04)								
]2.1;3.5]	2.8	1,742	1,762.0	0.99 (0.94, 1.04)	3.4	34	36.0	0.95 (0.65, 1.32)				
]3.5;5.4]	4.2	902	866.0	1.04 (0.97, 1.11)	4.6	922	937.6	0.98 (0.92, 1.05)				
]5.4;8.8]	6.3	89	93.1	0.96 (0.77, 1.18)	6.8	1,318	1,320.3	1.00 (0.95, 1.05)	7.9	411	405.2	1.01 (0.92, 1.12)
]8.8;11.2]					9.8	275	246.5	1.12 (0.99, 1.26)	9.9	653	644.9	1.01 (0.94, 1.09)
]11.2;13.4]					11.9	82	86.1	0.95 (0.76, 1.18)	12.1	353	361.6	0.98 (0.88, 1.08)
> 13.4					14.2	15	19.5	0.77 (0.43, 1.27)	16.0	437	442.4	0.99 (0.90, 1.08)
SIR by 1 mSv.year				1.02 (0.98, 1.07)				1.01 (0.99, 1.03)				1.00 (0.98, 1.01)

RNCE: National Registry of Childhood Cancers; Mean: mean weighted by the expected number of cases over 1990-2009; O: observed number of cases; E: expected number of cases; SIR (95% CI): standardized incidence ratio and its 95% confidence interval estimated by Poisson regression models for trend analyses, with Byar's approximation otherwise.

[a] Estimated cumulative exposure. The cutoffs of the categories of exposure are based on the distribution of the expected number of cases at the following percentiles p20%, p40%, p60%, p80%, p90%, p95%.

Table S4. Cumulative red bone marrow dose associated to radon and gamma radiation, and risk of ALL and AML in children under 15 years old, RNCE 1990-2009

Type of leukemia	ALL (N=7,434)				AML (N=1,465)			
	Mean	O	E	SIR (95% CI)	Mean	O	E	SIR (95% CI)
**Radon (mSv)** [a]								
≤ 2.5	0.9	6,630	6,624.1	1.00 (0.98, 1.03)	0.8	1,279	1,264.0	1.01 (0.96, 1.07)
]2.5;5]	3.3	655	655.8	1.00 (0.92, 1.08)	3.4	149	158.9	0.94 (0.79, 1.10)
]5.0;7.5]	5.9	100	108.4	0.92 (0.75, 1.12)	6.0	24	27.9	0.86 (0.55, 1.28)
>7.5	8.6	49	45.7	1.07 (0.79, 1.42)	8.6	13	14.3	0.91 (0.49, 1.56)
SIR by mSv				1.00 (0.98, 1.03) [b]				0.96 (0.91, 1.01)
**Gamma radiation (mSv)** [a]								
≤ 2.5	1.7	797	822.0	0.97 (0.90, 1.04)	1.5	417	412.5	1.01 (0.92, 1.11)
]2.5;5.0]	3.7	2,431	2,417.5	1.01 (0.97, 1.05)	3.5	254	264.3	0.96 (0.85, 1.09)
]5.0;7.5]	6.1	1,602	1,622.1	0.99 (0.94, 1.04)	6.2	195	186.5	1.05 (0.90, 1.20)
]7.5;10.0]	8.7	1,014	993.7	1.02 (0.96, 1.09)	8.8	180	178.5	1.01 (0.87, 1.17)
]10.0;15.0]	12.0	1,166	1,144.7	1.02 (0.96, 1.08)	12.1	285	293.0	0.97 (0.86, 1.09)
]15.0;20.0]	16.9	323	333.2	0.97 (0.87, 1.08)	16.9	105	97.9	1.07 (0.88, 1.30)
]20.0;25.0]	21.7	85	86.7	0.98 (0.78, 1.21)	21.6	25	27.6	0.91 (0.59, 1.34)
>25.0	25.4	16	14.1	1.13 (0.65, 1.84)	25.4	4	4.8	0.83 (0.22, 2.14)
SIR by mSv				1.01 (0.99, 1.02)				0.99 (0.97, 1.02)
**Total (mSv)** [a]								
≤ 2.5	1.8	577	600.9	0.96 (0.88, 1.04)	1.5	375	372.6	1.01 (0.91, 1.11)
]2.5;5.0]	3.8	2,114	2,118.5	1.00 (0.96, 1.04)	3.5	250	259.9	0.96 (0.85, 1.09)
]5.0;7.5]	6.1	1,570	1,536.7	1.02 (0.97, 1.07)	6.2	158	153.6	1.03 (0.88, 1.20)
]7.5;10.0]	8.6	998	1,027.1	0.97 (0.91, 1.03)	8.7	160	145.3	1.10 (0.94, 1.29)
]10.0;15.0]	12.2	1,335	1,315.9	1.02 (0.96, 1.07)	12.3	300	296.7	1.01 (0.90, 1.13)
]15.0;20.0]	17.0	542	540.0	1.00 (0.92, 1.09)	17.1	143	150.2	0.95 (0.80, 1.12)
]20.0;25.0]	22.0	205	207.6	0.99 (0.86, 1.13)	22.1	57	58.8	0.97 (0.74, 1.26)
>25.0	29.1	93	87.4	1.06 (0.86, 1.30)	29.1	22	27.9	0.79 (0.49, 1.19)
SIR by mSv				1.00 (1.00, 1.01)				0.99 (0.98, 1.01)

RNCE: National Registry of Childhood Cancers; ALL: acute lymphoid leukemia; AML: acute myeloid leukemia Mean: mean weighted by the expected number of cases over 1990-2009; O: observed number of cases; E: expected number of cases; SIR (95% CI): standardized incidence ratio and its 95% confidence interval estimated by Poisson regression models for trend analyses, with Byar's approximation otherwise.

[a] Estimated cumulative RBM dose. The cutoffs are categories of 2.5 mSv up to 10.0 mSv and 5.0 mSv above 10.0 mSv

[b] p value of the test of departure from linearity between 0.01 and 0.05.

Table S5. Exposure to radon and gamma radiation, and risk of acute leukemia in children under 15 years old, Geocap study 2002-2007

		All AL (N=2,763)		ALL (N=2,283)		AML (N=418)	
	Controls	Cases	OR (95%CI)	Cases	OR (95%CI)	Cases	OR (95%CI)
**Radon exposure (Bq/m^3)**							
[12.8 ; 37.7]	6,004	530	1.00 (Ref.)	423	1.00 (Ref.)	96	1.00 (Ref.)
]37.7 ; 49.1]	5,996	526	1.02 (0.90, 1.16)	436	1.07 (0.93, 1.23)	82	0.85 (0.63, 1.14)
]49.1 ; 62.8]	6,005	587	1.13 (1.00, 1.28)	486	1.17 (1.02, 1.35)	86	0.92 (0.68, 1.23)
]62.8 ; 90.5]	5,996	575	1.11 (0.98, 1.26)	491	1.19 (1.04, 1.37)	71	0.74 (0.55, 1.01)
]90.5 ; 801.3]	5,999	545	1.04 (0.91, 1.18)	447	1.07 (0.93, 1.23)	83	0.88 (0.65, 1.18)
OR by 100 Bq/m^3	30,000	2,763	0.98 (0.90, 1.07)	2,283	0.99 (0.90, 1.09) [b]	418	0.90 (0.71, 1.13)
**Gamma radiation exposure (nSv/h)** [a]							
[62.4 ; 76]	5,946	530	1.00 (Ref.)	426	1.00 (Ref.)	91	1.00 (Ref.)
]76 ; 86]	5,945	546	1.05 (0.92, 1.19)	456	1.09 (0.95, 1.25)	79	0.88 (0.65, 1.19)
]86 ; 98.3]	5,948	598	1.14 (1.01, 1.29)	492	1.16 (1.02, 1.33)	91	1.03 (0.77, 1.38)
]98.3 ; 118.5]	5,945	525	1.01 (0.89, 1.14)	431	1.03 (0.90, 1.19)	82	0.91 (0.68, 1.24)
]118.5 ; 254.7]	5,946	545	1.02 (0.90, 1.16)	463	1.08 (0.94, 1.24)	71	0.79 (0.57, 1.07)
OR by 10 nSv/h	29,730	2,744	1.00 (0.98, 1.01)	2,268	1.00 (0.98, 1.02)	414	0.97 (0.93, 1.01)

AL: childhood acute leukemia; ALL: acute lymphoid leukemia; AML: acute myeloid leukemia; OR (95%CI): Odds ratio and its 95% confidence interval estimated by logistic regression adjusted for year of age

[a] 270 missing values for controls, 19 for AL, 15 for ALL, 4 for AML

[b] p value of the test of departure from linearity between 0.01 and 0.05
